# Coxsackievirus A6 and Hand, Foot, and Mouth Disease, Finland

**DOI:** 10.3201/eid1509.090438

**Published:** 2009-09

**Authors:** Riikka Österback, Tytti Vuorinen, Mervi Linna, Petri Susi, Timo Hyypiä, Matti Waris

**Affiliations:** University of Turku, Turku, Finland (R. Österback, T. Vuorinen, P. Susi, T. Hyypiä, M. Waris); Central Hospital of Seinäjoki, Seinäjoki, Finland (M. Linna)

**Keywords:** HFMD, coxsackievirus A, onychomadesis, enterovirus, picornavirus, vesicular rash, nail shedding, Finland, viruses, dispatch

## Abstract

During fall 2008, an outbreak of hand, foot, and mouth disease (HFMD) with onychomadesis (nail shedding) as a common feature occurred in Finland. We identified an unusual enterovirus type, coxsackievirus A6 (CVA6), as the causative agent. CVA6 infections may be emerging as a new and major cause of epidemic HFMD.

Hand, foot, and mouth disease (HFMD) is a common childhood illness characterized by fever and vesicular eruptions on hands and feet and in the mouth (Figure 1). It is caused by members of the family *Picornaviridae* in the genus *Enterovirus*. Complications are rare, but pneumonia, meningitis, or encephalitis may occur. Outbreaks of HFMD have been mainly caused by 2 types of enterovirus A species, coxsackievirus (CV) A16 (CVA16) or enterovirus 71 (*1*). Some outbreaks have been associated with CVA10, but only sporadic cases involving other members of the enterovirus A species have been reported (*2**,**3*).

**Figure 1:**
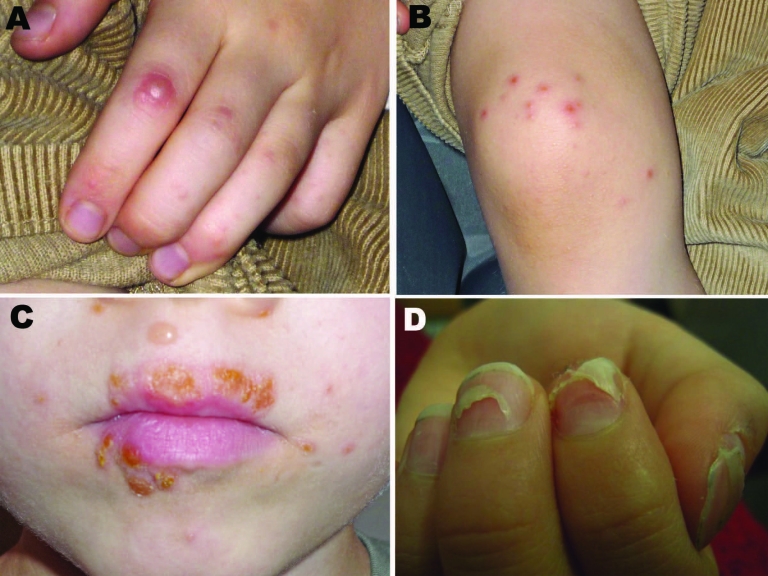
Vesicular eruptions in A) hand, B) foot, and C) mouth of a 6.5-year-old boy from Turku, Finland, with coxsackievirus (CV) A6 infection. Several of his fingernails shed 2 months after the pictures were taken. D) Onychomadesis in a 10-year-old boy from Seinäjoki, Finland, 2 months after hand, foot and mouth disease with CVA6 infection. Photographs courtesy of H. Kujari (A–C) and M. Linna (D).

During fall 2008, a nationwide outbreak of HFMD occurred in daycare centers and schools in Finland, starting in August and continuing at least until the end of the year and possibly into the following year. From vesicle fluid specimens of hospitalized children, we identified the etiologic agent as coxsackievirus A6.

## The Study

In August 2008, vesicle fluid specimens were collected from 2 children and 1 parent with HFMD at the Central Hospital of Seinäjoki, Southern Ostrobothnia. Specimens were sent to the Department of Virology, University of Turku, for identification of the causative agent. After detection of CVA6 in these index cases, the virus was also found in specimens obtained from the Pirkanmaa Hospital District (Tampere), Turku University Hospital (Turku), Pori Central Hospital (Pori), and *Central-Ostrobothnia Central Hospital (Kokkola)* (Table).

**Table:** Laboratory findings in clinical specimens and epidemiologic data for patients with CVA6 infections, Finland, 2008*

City or place, identification	Sampling date	Sex/age, y	Specimen type	Disease or signs	CVA6-VP1 RT-PCR	VP1 sequence	5′ NCR sequence
Seinäjoki							
Fin/Se8717	2008 Aug	M/1.3	Vesicle fluid	HFMD	Pos	CVA6	CVA6
Fin/Se8781	2008 Aug	F/34	Vesicle fluid	HFMD	Pos	CVA6	CVA6
Fin/Se8841	2008 Aug	M/0.9	Vesicle fluid	HFMD	Pos	CVA6	CVA6
Fin/Se8865	2008 Aug	†	Feces	HFMD	Pos	NR	CVA6
Fin/Se8913	2008 Sep	M/10	Throat swab	HFMD	Pos	NR	CVA6
Fin/Se8925	2008 Sep	M/0.9	Throat swab	HFMD	Pos	NR	CVA6
Fin/Se8926	2008 Sep	†	Vesicle fluid	HFMD	Pos	CVA6	CVA6
Fin/Se8927	2008 Sep	F/1.3	Throat swab	HFMD	Pos	NR	CVA6
Fin/Se8928	2008 Sep	†	Vesicle fluid	HFMD	Pos	CVA6	CVA6
Fin/Se8931	2008 Sep	M/0.8	Throat swab	NA	Pos	NR	CVA6
Turku							
Fin/Tu8859	2008 Sep	M/5.8	Throat swab	Fever, tonsillitis	Pos	CVA6	CVA6
Fin/Tu8866	2008 Sep	M/3.1	Throat swab	HFMD	Pos	NR	CVA6
Fin/Tu81027	2008 Oct	M/1.8	Throat swab	HFMD	Pos	NR	CVA6
Fin/Tu81042	2008 Oct	M/1.8	Throat swab	Fever, eczema	Pos	NR	CVA6
Fin/Tu81038	2008 Oct	F/2.2	Throat swab	Fever, seizure	Pos	NR	CVA6
Fin/Tu81274	2008 Nov	M/6.5	Vesicle fluid	HFMD	Pos	ND	CVA6
Fin/Tu81309	2008 Dec	F/3.7	Vesicle fluid	HFMD	Pos	ND	CVA6
Fin/Tu81321	2008 Dec	F/1.2	Throat swab	HFMD	Pos	ND	NR
Fin/Tu963	2009 Jan	M/1.2	Throat swab	HFMD	Pos	ND	CVA6
Fin/Tu/IB	2009 Feb	F/5.7	Nail	Recent HFMD	Pos	ND	CVA6
Pori							
Fin/Po8959	2008 Oct	M/4.8	Throat	Fever, vomiting	Neg	ND	CVA6
Fin/Po81375	2008 Dec	M/0.5	Vesicle fluid	HFMD	Pos	ND	CVA6
Fin/Po81376	2008 Dec	†	Throat	HFMD	Pos	ND	CVA6
Fin/Po81324	2008 Dec	M/10	Feces	Fever, eczema	Pos	ND	NR
Fin/Po81325	2008 Dec	M/10	Feces	Fever, HFMD contact	Pos	ND	NR
Tampere							
Fin/Ta8966	2008 Sep	M/3.1	Tracheal aspirate	NA	Pos	NR	CVA6
Fin/Ta81145	2008 Sep	M/2.1	Feces	NA	Pos	NR	NR
Fin/Ta81074	2008 Oct	M/0.3	Tracheal aspirate	NA	Pos	ND	CVA6
Fin/Ta81125	2008 Oct	F/0.7	Vesicle fluid	HFMD	Pos	CVA6	CVA6
Fin/Ta81126	2008 Oct	M/0.3	Vesicle fluid	HFMD	Pos	NR	CVA6
Fin/Ta81216	2008 Nov	F/2.0	Throat swab	NA	Pos	ND	CVA6
Fin/Ta81252	2008 Nov	M/6.1	Throat swab	NA	Pos	ND	CVA6
Kokkola							
Fin/Ko937	2009 Jan	M/10	Vesicle fluid	HFMD	Pos	ND	CVA6

*CVA6, coxsackievirus A6; VP1, virus protein 1; RT-PCR, reverse transcriptase–PCR; NCR, noncoding region; HFMD, hand, foot, and mouth disease; pos, positive; neg, negative; NA, not available; ND, not done; NR, no result (VP1 sequencing was attempted without result). †Same patient as on the line above.

Nucleic acids were extracted from specimens by using the NucliSens EasyMag automated extractor (bioMèrieux, Boxtel, the Netherlands). When the extracts were analyzed for enteroviruses by using real-time reverse transcriptase–PCR (RT-PCR) specific for the 5′ noncoding region (NCR) of picornaviruses (*4*), amplicons with melting points indistinguishable from each other and typical to enteroviruses were obtained.

**Figure 2:**
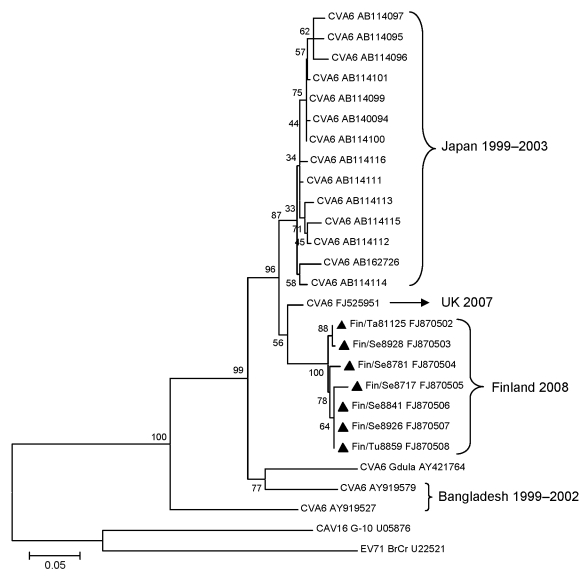
Phylogenetic analysis of coxsackievirus (CV) A6 partial (289 bp) viral protein 1 sequences showing the relationships between the recent clinical CVA6 samples isolated in Finland (triangles), selected CVA6 isolates from GenBank, and prototypes of CVA6, CVA16, and enterovirus (EV) 71. GenBank accession numbers are included. Scale bar indicates nucleotide substitutions per position.

To identify the enterovirus type in the specimens, RT-PCR, specific for a partial sequence of the viral protein 1 (VP1) region, was performed by using the COnsensus-DEgenerate Hybrid Oligonucleotide (CODEHOP) Primers (bioinformatics.weizmann.ac.il/blocks/codehop.html) (*5*). The amplicons were separated by agarose gel electrophoresis, purified with the QIAquick PCR Purification Kit (QIAGEN, Hilden, Germany), and sequenced in the DNA Sequencing Service Laboratory of the Turku Centre for Biotechnology. The virus type in the 3 index specimens, 3 samples of vesicular fluids, and 1 throat swab was successfully identified with sequencing and BLAST (www.ncbi.nlm.gov/BLAST) analysis as CVA6. Phylogenetic relationships of the sequences were examined by using CVA6 (Gdula strain), CVA16 (G10), and enterovirus 71 (BrCr) prototype strains as well as selected clinical CVA6 isolates obtained from GenBank. Sequence alignments were generated with the ClustalW program (www.ebi.ac.uk/clustalw), and the phylogenetic tree was computed by using the Jukes-Cantor algorithm and the neighbor-joining method. Phylogenetic analyses were conducted by using MEGA4 software (www.megasoftware.net) and the bootstrap consensus tree inferred from 1,000 replicates (*6*) (Figure 2).

Phylogenetic analysis placed all CVA6 strains from the HFMD outbreak in 1 cluster (97%–100% identity), whereas the nucleotide identities between those isolates and CVA6 prototype strains Gdula, CAV16, G-10, and enterovirus 71 BrCr were 82.5%–83.2%, 55.6%–56.6%, and 55.6%–57.3%, respectively. The closest preceding CVA6 strain was isolated from cerebrospinal fluid in the United Kingdom in 2007 and had 92%–94% nucleotide identity with the strains described here (*7*).

To improve the detection of the novel CVA6 strains in clinical specimens, we designed specific VP1 primers from the aligned sequences. CVA6vp1 reverse primer (5′-ACTCGCTGTGTGATGAATCG-3′) and CVA6vp1 forward primer (5′-CGTCAAAGCGCATGTATGTT-3′) generated a 199-bp amplicon. First, cDNA was synthesized in a 20-μL reaction mixture containing 1 μmol/L CVA6vp1 reverse primer, 2.5 mmol/L of each dNTP, 20 U RevertAid H Minus M-MuLV reverse transcriptase (Fermentas, St. Leon-Rot, Germany), reaction buffer (Fermentas), 4 U RiboLock RNase inhibitor (Fermentas), and 5 μL RNA incubated at 42ºC for 1 h. Then, 5 μL of cDNA was added to 20 μL of master mixture containing 0.4 μmol/L each of the CVA6vp1 primers and Maxima SYBR Green qPCR Master Mix (Fermentas). PCR with melting curve analysis was performed in a Rotor-Gene 6000 real-time instrument (Corbett Research, Mortlake, Victoria, Australia) by using the following cycling conditions: initial denaturation at 95ºC for 10 min, 45 cycles at 95ºC for 15 s, 60ºC for 30 s, and at 72ºC for 45 s, followed by generation of melting curve from 72°C to 95°C with temperature increments of 0.5°C/s. Partial 5′ NCR sequence of the strains in clinical specimens was determined as described (*4*) and compared with the known sequences by using BLAST (http://blast.ncbi.nlm.nih.gov/Blast.cgi).

During autumn 2008, a total of 47 acute-phase specimens, including 12 vesicle fluid samples, 23 throat swabs, 2 tracheal aspirates, 5 fecal samples, and 5 cerebrospinal fluid specimens from 43 patients yielded amplicons with similar melting points as the originally identified CVA6 strains in 5′ NCR RT-PCR. All specimens were subjected to the specific CVA6-VP1 real-time RT-PCR, and a positive result was obtained for 11 vesicle fluid samples, 14 throat swabs, 2 tracheal aspirates, and 4 fecal samples (Table). The virus in 1 throat swab was identified as CVA6 from the result of 5′ NCR sequencing alone. None of the CVA6-positive specimens were positive by an RT-PCR assay with CVA16- and EV71-specific primers (*8*). Attempts to cultivate the virus from 8 CVA6 RT-PCR-positive specimens were unsuccessful, whereas the prototype strain could be propagated in rhabdomyosarcoma cells.

Onychomadesis was 1 characteristic feature in patients during this HFMD outbreak; parents and clinicians reported that their children shed fingernails and/or toenails within 1–2 months after HFMD (Figure 1). Only a few published reports of nail matrix arrest in children with a clinical history of HFMD exist in the medical literature (*9**–**11*). We obtained shed nails from 2 siblings who had HFMD 8 weeks before the nail shedding. The nail fragments were stored at –70°C for a few weeks and treated with proteinase K before nucleic acid extraction. The extracts were enterovirus positive in 5′ NCR RT-PCR. The virus in one of them was identified as CVA6 by the specific RT-PCR and yielded a 5′ NCR sequence that was similar to the novel CVA6 strains.

## Conclusions

Enterovirus CVA6 was a primary pathogen associated with HFMD during a nationwide outbreak in Finland in autumn of 2008. HFMD epidemics have primarily been associated with CVA16 or enterovirus 71 infections; those caused by enterovirus 71 have occurred more frequently in Southeast Asia and Australia in recent years (*12*). Reportedly, CVA10 has been found in minor outbreaks; other coxsackievirus A types have been found in only sporadic cases of HFMD (*2*,*3*). In general, CVA6 infections have been seldom detected and mostly in association with herpangina (*13**,**14*). In Finland, CVA6 has been identified only on 4 occasions over 8 years during enterovirus surveillance from 2000 to 2007 (*15*).

Although the CODEHOP primers were elementary for rapid genotyping of the novel CVA6 strains, we identified more viruses with the designated CVA6-VP1 specific primers. Onychomadesis was a hallmark of this HFMD outbreak. To our surprise, we detected CVA6 also in a fragment of shed nail. The same virus could have given rise to the outbreak in Spain in 2008 (*10*). Supposedly, virus replication damages nail matrix and results in temporary nail dystrophy. Whether nail matrix arrest is specific to CVA6 infections remains to be shown. This study demonstrates that CVA6, in addition to CVA16 and enterovirus 71, may be emerging as a primary cause of HFMD.
